# Supplementary material for: Degradation of methylene blue by natural manganese oxides: kinetics and transformation products
Source: R Soc Open Sci. 2019 Jul 10;6(7):190351. doi: 10.1098/rsos.190351 (PMC6689640; doi:10.1098/rsos.190351)
Supplement: Supplementary informantion [file rsos190351supp1.docx]

**Supplementary Information for**

# Degradation of methylene blue by natural manganese oxides: Kinetics and transformation products

Shuangxi Zhou, Zhiling Du, Xiuwen Li, Yunhai Zhang, Yide He*, Yongjun Zhang*

School of Environmental Science and Engineering, Nanjing Tech University, Nanjing, P.R. China

Corresponding author: Pu Zhu Nan Lu 30, 211800, Nanjing, P.R. China

Email: y.zhang@njtech.edu.cn, heyd@njtech.edu.cn

(b)


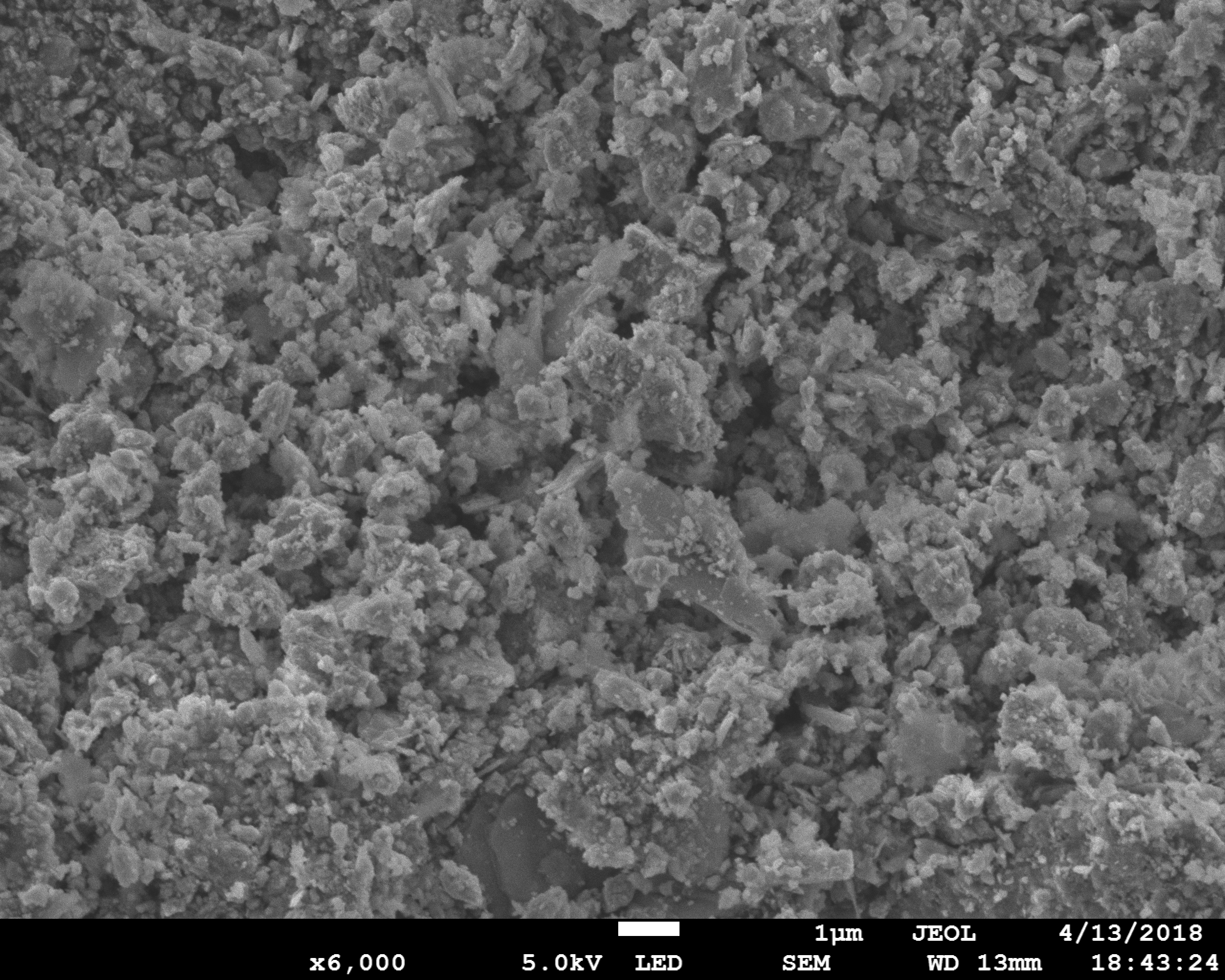


**Figure S1.**SEM image of MnOx

The method for the measurement of pH_pzc_:

The point zero charges (pHpzc) value of MnOx was measured by a traditional potentiometric acid–base titration method which was conducted in 250ml flask with a series of 100 mL NaCl (0.01 M) solutions, and the pH values were adjusted from 2.0 to 10.0 using 0.1 M HCl or 0.1 M NaOH. After that, 0.15 g MnOx was added and the mixture was shaked in a thermostatic shaker at 120 rpm (30 ℃) for 48 h. The initial pH (pH_initial_) and final pH (pH_final_) were recorded. The pHpzc value is obtained from the plot ΔpH = (pH_initial_ - pH_final_) versus initial pH_initial_. The intersection of the curve with the straight line is the point of pH_pzc_.

**Figure S2.**The zero point of MnOx measured by a traditional potentiometric acid–base titration method

**Table∣S1** Chemical bonds energies of methylene blue molecule [1]

| Molecule bonds | Bond energy (kcal/mol) |
| --- | --- |
| CH_3_-N(CH_3_)C_6_H_5_ | 70.8 |
| N(CH_3_)_2_C_6_H_5_ | 93.2±2.5 |
| C_6_H_5_-N-C_6_H_5_ | 87.4 |
| C_6_H_5_-S-C_6_H_5_ | 76±2 |
| C_6_H_5_-NH_2_ | 102.6±1.0 |

## References:

1. Luo, Y. R., 2005 Handbook of Bond Dissociation Energies in Organic Compounds. Science Press. Beijing.
